# Recycling the LDL receptor to combat atherosclerosis

**DOI:** 10.18632/aging.101681

**Published:** 2018-11-27

**Authors:** Dyonne Vos, Jan Albert Kuivenhoven, Bart van de Sluis

**Affiliations:** 1 Department of Pediatrics, Molecular Genetics Section, University of Groningen, University Medical Center Groningen, Groningen 9713 AV, The Netherlands.

**Keywords:** atherosclerosis, LDLR, endosomal sorting complex, hypercholesterolemia, recycling

Atherosclerosis, the pathology underlying atherosclerotic cardiovascular disease (ASCVD), is an ageing disorder. It starts in early adolescence but the clinical consequences (normally) appear beyond the age of 55 and 65 in men and women, respectively. A major risk factor for ASCVD is high plasma levels of low-density lipoprotein cholesterol (LDL-c), which gradually increases with age [1]. It has been indicated that the causal effect of LDL-c on ASCVD risk is not only determined by the absolute quantity but also by the cumulative duration of exposure to LDL-c [2].

**Figure 1:**
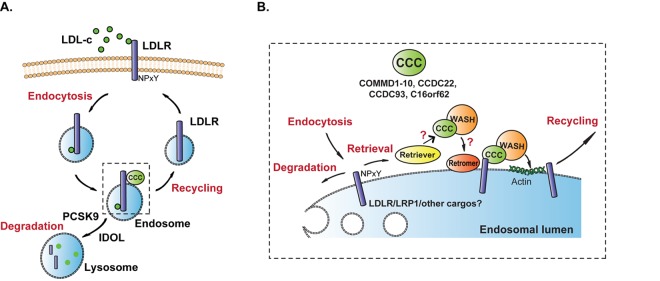
**Working model of the endosomal recycling mechanism of LDLR.** (**A**) LDLR and LDL-c are both endocytosed. LDLR can be directed to the lysosome for proteolysis, which is promoted by the proteins PCSK9 and IDOL, or it can be recycled and transported from the endosomes back to the cell surface. (**B**) Magnification of inset in **A**. At the endosomes LDLR is retrieved from the degradation pathway and directed to the recycling pathway. The CCC complex is formed by the COMMD proteins (COMMD1-10) and the core proteins (CCDC22, CCDC93 and C16orf62). The recycling is facilitated by the CCC and WASH complexes via a direct interaction between COMMD1 and LDLR. COMMD1-LDLR interaction is dependent on the NPxY domain in the cytosolic tail of LDLR. Loss of any CCC component (either a COMMD protein or a core component) destabilizes the complete CCC complex and results in diminished recycling of LDLR. The recruitment of the CCC and WASH complexes to the endosomes is partially dependent on retromer (VPS35, VPS29, VPS26), but it is still unclear whether LDLR recycling is retromer-dependent. Recently, retriever (DSCR3/VPS26c-C16orf62/VPS35L-VPS29) has been identified as a complex that retrieves transmembrane proteins from degradation pathways. Retriever is associated with the CCC complex but its exact function and its molecular organization in the CCC-WASH axis and in LDLR trafficking is still unclear. Like LDLR, also the recycling of the lipoprotein receptor LRP1 is dependent on the CCC complex, however, which other transmembrane proteins are recycled by the CCC complex remains to be determined.

The LDL receptor (LDLR) is the major receptor for the hepatic uptake of plasma LDL-c and mutations in *LDLR* cause familial hypercholesterolemia and premature atherosclerosis. At the cell surface, LDL binds to LDLR and both are internalized via clathrin-coated pits and transported to the endosomes (Figure 1, panel A). Upon entering the endosomes, LDLR - together with LDL- can either be directed to the lysosomes for degradation or the receptor can be retrieved from this degradation pathway and transported back to the cell surface for a new round of LDL binding and uptake (Figure 1, panel A). Although the proteins that determine the fate of LDLR towards the degradation pathway, i.e. Proprotein Convertase Subtilisin/Kexin type 9 (PCSK9) and inducible degrader of LDLR (IDOL), have been well characterized [3], the proteins that are responsible for the recycling of LDLR from the endosomes back to the cell surface have until shortly been a mystery. In 2016, we found that the copper metabolism MURR1 domain-containing protein (COMMD) 1 facilitates the recycling of LDLR back to the cell surface in mice and dogs [4].

COMMD1, a member of the COMMD family of proteins that is comprised of ten members (COMMD1-10), was first identified as an essential protein for hepatic copper homeostasis [5]. COMMD1 assembles with the coiled-coil domain–containing proteins (CCDC) CCDC22 and CCDC93 and the chromosome 16 open reading frame 62 (C16orf62) protein the CCC (COMMD/CCDC22/CCDC93) complex (Figure 1, panel B). It has recently been shown that the CCC complex is associated with the Wiskott–Aldrich syndrome protein and SCAR homologue (WASH) complex to mediate the endosomal trafficking of the copper transporting protein ATP7A. Using COMMD1 deficient mouse embryonic fibroblasts (MEFs) and primary hepatocytes, we showed that the CCC complex is also required for the endosomal transport of LDLR. Loss of COMMD1 led to accumulation of LDLR in endosomes and reduced levels of LDLR at the cell surface, which in turn resulted in diminished LDL uptake [4]. This compromised LDLR recycling and LDL uptake likely underlies the hypercholesterolemic phenotype in mice and dogs lacking COMMD1 in hepatocytes [4]. Interestingly, ablation of the WASH complex in MEFs also impairs endosomal LDLR trafficking [4], which supports the notion that the CCC complex acts in concert with the WASH complex and indicates that they are both required for the recycling of LDLR. It is, however, not yet known whether inactivation of the WASH complex in the liver also leads to hypercholesterolemia.

Recently, we unexpectedly identified that the integrity of the CCC core complex consisting CCDC22, CCDC93 and C16orf62 not only relies on COMMD1 [4] but also on other members of the COMMD family [6]. Hepatic deletion of *Commd6* or *Commd9* in mice also strongly reduces the hepatic levels of the CCC core subunits (CCDC22, CCDC93 and C16orf62), accompanied by elevated plasma LDL-c and increased atherosclerosis [4,6]. Hepatic deficiency of any COMMD protein not only affects the protein levels of the CCC core components but also the levels of all other members of the COMMD family. A very similar effect on the protein levels of the CCC complex and COMMDs was also observed upon CRISPR-mediated ablation of *Ccdc22* in mouse livers [6]. These data indicate that all COMMD proteins participate in shaping the CCC complex, but whether all COMMDs form together one large protein complex or multiple subcomplexes with the CCC core components remains unclear. In addition, the function of the CCC complex in the endosomal trafficking machinery and why it is decorated with ten different COMMD proteins has still to be determined. One of the current hypotheses is that the COMMD proteins equip the endosomal sorting machinery with an extra layer of specificity in recognition and transport of transmembrane proteins.

The observation that patients with X-linked intellectual disability (XLID) and Ritscher-Schinzel Syndrome - caused by mutations in *CCDC22* and the *WASHC5* (encoding for WASH component WASHC5/strumpellin), respectively - are hypercholesterolemic [4] illustrates that this endocytic LDLR recycling machinery is conserved across species. Therefore, it would be of great interest to assess whether common genetic variants in components of the CCC and WASH complexes exist that are related to plasma LDL-c levels and CVD risk in humans, and ultimately, whether this recycling pathway can be therapeutically improved to lower plasma LDL-c levels and eventually CVD risk.

Besides CCC and WASH, other multiprotein complexes have been reported to coordinate the endosomal transport of transmembrane proteins [7]. One of the best studied complexes is the retromer complex. Retromer, consisting of the proteins VPS35, VPS26 and VPS29, acts in concert with WASH to coordinate the transport of a wide range of transmembrane proteins from the endosomes to plasma membrane or to the *trans*-Golgi network [7]. Although it has been well-established that the recruitment of the WASH complex to the endosome relies on the retromer subunit VPS35, a recent study has shown that WASH can also be recruited to endosomal membranes in a retromer-independent manner (Figure 1, panel B) [7], but whether retromer is required for WASH-mediated LDLR recycling has yet to be determined.

In this same study, the authors described a novel protein complex called ‘retriever’ (DSCR3/VPS26C-C16orf62/VPS35L-VPS29) [7], which shares structural similarity with retromer. The endosomal association of retriever is dependent on its interaction with the CCC complex and it acts as a separate complex in the CCC-WASH axis in a retromer-independent manner [7]. Interestingly, the retriever component C16orf62 (also referred to as VPS35L) was initially identified as a component of the CCC complex. Our recent studies strongly support this original model, as the protein level of C16orf62, but not the retriever component VPS29, was blunted upon depletion of either CCDC22 or any COMMD protein [4,6]. These data suggest that C16orf62 participates in the formation of a stable CCC complex and does not form a distinct multiprotein complex in the CCC-WASH axis as suggested in this study [7]. This notion is further supported by recent systems biology studies [8], which have suggested that the CCC complex and retriever form a large evolutionary conserved multiprotein complex called the COMMander [8]. Altogether, these contradicting data warrant more research to understand the molecular organization and functional role of retriever and CCC complex in endosomal transport of transmembrane proteins. Although, the transmembrane proteins that are dependent on the WASH and CCC complexes have yet to be determined, our recent study revealed that the endosomal trafficking of the low-density lipoprotein receptor-related protein 1 (LRP1) also relies on the CCC complex [6]. LRP1 is a member of the LDLR family and like many other members of the LDLR family (such as LDLR, VLDLR, SORL1, and ApoER2) contains a NPxY motif within its cytoplasmic tail. This motif is important for COMMD1 to bind to LDLR, thus it is very conceivable that the recycling of other LDLR family members are also dependent on the CCC complex. Furthermore, since the CCC complex is ubiquitously expressed it is of great interest to study the physiological contribution of this pathway in other tissues, such as adipose tissue, brain, kidney, and intestine.

Taken together, these recent studies provided valuable insights into the molecular mechanism by which LDLR is recycled back to the cell surface. This pathway is conserved across species and is needed to maintain normal plasma LDL-c levels.
